# Aquatic Plant Diversity in Italy: Distribution, Drivers and Strategic Conservation Actions

**DOI:** 10.3389/fpls.2018.00116

**Published:** 2018-02-13

**Authors:** Rossano Bolpagni, Alex Laini, Chiara Stanzani, Alessandro Chiarucci

**Affiliations:** ^1^Department of Chemistry, Life Sciences, and Environmental Sustainability, University of Parma, Parma, Italy; ^2^Department of Biological, Geological and Environmental Sciences, University of Bologna, Bologna, Italy

**Keywords:** macrophytes, vascular plants, spatial distribution, environmental drivers, freshwater ecosystems, human impacts

## Abstract

Italy is recognized as one of the prominent hot spot areas for plant diversity at regional and global scale, hosting a rich range of ecosystems and habitat types. This is especially true considering aquatic habitats, which represent a major portion of the total water surfaces in the Mediterranean region. Nevertheless, only a scant attention was paid to clarify the species richness of aquatic plant and its contribution to the total diversity at the country scale, despite such plants are seriously threatened at multiple scales. This paper provided the first comprehensive inventory of aquatic plants at the whole country scale, collecting data on species’ distribution, trends, and explanatory determinants of species richness. We confirmed the key contribution of Italy to the regional and global aquatic plant diversity with a total of 279 species recorded since 2005, equal to the 88.5%, 55.9% and ∼10% of the richness estimated at European/Mediterranean, Palearctic and global scale, respectively. Ten species are considered extinct in the wild [among which *Aldrovanda vesiculosa* L., *Caldesia parnassifolia* (Bassi ex L.) Parl., *Helosciadium repens* (Jacq.) W.J.D. Koch, and *Pilularia globulifera* L.], four were doubt [among which *Luronium natans* (L.) Raf., *Utricularia intermedia* Hayne, and *U. ochroleuca* R.W. Hartman.], and eight were erroneously reported in the past, among which *Isoëtes lacustris* L., *Myosotis rehsteineri* Wartm., and *Ranunculus aquatilis* L. Only 18 species – mainly helophytes (14) – were present in all the 20 Italian regions, whereas hydrophytes showed most scanty regional frequencies. Temperature, latitude, area and water resources availability are the main drivers of aquatic plant spatial arrangement and diversity. Furthermore, the number of inhabitants per km^2^ well described the number of “lost species” since 2000. The findings of the present survey call for an urgent elaboration of large-scale strategies to ensure the survival of aquatic plants, stressing on multiple functions played by aquatic plants in supporting national economy and human well-being. In this context, Italy can play a fundamental role guaranteeing temporary refuge for projected or expected species migrations along latitude and longitude gradients. Besides, in hyper-exploited landscapes man-made water bodies can further enhance the achievement of minimum conservation targets.

## Introduction

Italy is the focal axis of the Mediterranean basin region: a biodiversity hotspot that host a very rich range of ecosystems and habitats, and about the 10% of the world’s higher plants (Médail and Quézel, 1999; Thompson, 2005; Cuttelod et al., 2008). Within this region, the Italian peninsula is among major centers of species richness, with a high number of endemic species, especially in terms of vascular plants (Rossi et al., 2013; Peruzzi et al., 2014). This is due to the presence of multiple key alert areas for plant diversity (e.g., Maritime and Ligurian Alps, Tyrrhenian Islands), acting both as refuge and exchanging floral areas, supporting active plant speciation (Médail and Quézel, 1999). This is due not only through the preservation of genotypes during glacial periods, but also thanks to the intensity and accumulation of multiple processes in a patchy landscape based on a complex topographic matrix (Nieto Feliner, 2011).

Bilz et al. (2011) and Chappuis et al. (2012) have recently assessed the key contribution of Italy in maintaining a prevalent share of aquatic plants at the European and Mediterranean scale. In fact, Italy incorporates a large part of the total water bodies in the region, encompassing ∼80% of the deep lakes within the Mediterranean coastal areas and 42% of the area occupied by deep lakes. In addition, its Northern corner includes a large part of the Alpine chain and the Northern Apennine sector that feed a complex system of rivers and lakes, and groundwater aquifers (Azzella et al., 2014; Bocchiola, 2014).

Aquatic plants play complex interconnected functions, including – among others – C and nutrient cyclization, sediment and riparian sectors stabilization, and the provision of food and habitats for a variety of animal species (Chambers et al., 2008; O’Hare et al., 2017). They act as “engineering species” (Bouma et al., 2010; Bolpagni et al., 2015), and their disappearance causes drastic effects on trophic and functional status of the habitats within water bodies (Scheffer et al., 2003; Soana and Bartoli, 2014). Nevertheless, aquatic plants remain often unrecognized in broad-scale investigations, a condition that can lead to wrong or to conflicting evaluations in analyzing their current spatial patterns and rarity (Alahuhta et al., 2017 and references therein). In this context, Italy thanks to its great heterogeneity in hydro-ecoregions and habitats, covering a wide latitudinal and altitudinal range, is of focal importance for aquatic plant conservation at the continental and global scales. This imposes a number of challenges in supporting effective management plans, including the streamlining of the available data concerning the current distribution of aquatic plants, the consistency of their populations, and the impacts they suffer from human activities.

Hence, Italy is subjected to extremely high rates of human perturbations, with huge effects on plant diversity, especially in lowland areas where the collapse of the traditional agro-sylvo-pastoral system, the land use changes and the soil artificialization have led to a major loss of natural and semi-natural patches (Falcucci et al., 2007; Bolpagni and Piotti, 2015, 2016). Among others, in lowlands aquatic and riparian vegetation has shown a rapid and constant decline due to the impairment of river discharge regimes and land reclamation (Bolpagni et al., 2013). Therefore, species-poor, ruderal or simplified plant communities have replaced pristine complex aquatic vegetation (Bolpagni and Piotti, 2015, 2016). As a result, frequently, less-demanding aquatic primary producers, such as cyanobacteria, soft-bodied benthic algae dominate remnant aquatic ecosystems (Scheffer et al., 2003; Barrett et al., 2010).

In the recently updated Red List of Italian Flora (focused on the Policy Species and the extremely threatened plants), Rossi et al. (2013) have largely stressed the critical conservation status of plant species ecologically connected with inland water ecosystems. Currently, the only Italian policy plant species that are extinct in the wild are two lowland obligated aquatic plants [*Aldrovanda vesiculosa* L., and *Caldesia parnassifolia* (Bassi ex L.) Parl.], and – furthermore – the 50% of the critically endangered “probably extinct” species (=six species) are hydrophytes (Rossi et al., 2013; Ercole and Giacanelli, 2014).

Additionally, over the last years frequent significant anomalies both in terms of thermal and rainfall regimes were detected, especially in northern sectors, reinforcing the general awareness on the extreme vulnerability of Italian peninsula to climate change (Hoff, 2013; Marchina et al., 2017). Despite this, no systematic inventory was made to deepen the spatial patterns of aquatic species across Italy, and to acquire information on their trends and main drivers.

In this work, the hydro- and hygrophilous plant diversity was investigated at regional scale at the national scale. In agreement with Chappuis et al. (2012) and Alahuhta et al. (2017), a strong dependence of aquatic plant richness on environmental heterogeneity, mainly supported by climate-related restrictions (altitudinal-grown limitation) and water quality gradients (at low altitudes) was hypothesized. Furthermore, the amount of water bodies potentially available for colonization is expected to have a central role in driving aquatic plant geographical distribution. In this context, we aimed to add new insights on: (1) an up-to-date species richness assessment of aquatic plant flora and its regional distribution; (2) the incidence of hydrophytes and not-obligate aquatic plants (e.g., helophytes or amphibian plants with a predominant hydrophytic phase) in the aquatic plant flora; and (3) the main determinants of aquatic plant diversity, focusing on environmental drivers (including climate, habitat conditions and human impacts).

## Materials and Methods

### Study Area

All the 20 administrative regions that constitute the territory of Italy were investigated, considering a total area of 301,338 km^2^. Among Mediterranean countries, Italy is the richer in water resources, it counts 69 natural lakes equal to or larger than 0.5 km^2^, 183 artificial basins larger than 1 km^2^, and more than 230 rivers and streams of particular relevance: 58 exceeding 100 km in length, and 75 with average daily discharges greater than 10 m^3^ s^-1^. Nevertheless, since Roman age almost all of the national wetland complexes and the riverine landscapes have been progressively reclaimed, and transformed into productive lands, especially in lowlands (Barone et al., 1985; de Haas, 2015). Recent estimates indicate a total area occupied by wetlands (including ponds and small shallow lakes < 3 ha) and riverscapes of about 6,000 km^2^, just over the 2% of the national territory, compared to the pristine wetland area in Roman age estimated to 30,000 km^2^ (equal to the 10.0% of the area). At the same time, the quality status of surface waters is far from the objectives set by the Water Framework Directive (WFD), with more than half of the water bodies being in less than good ecological status or potential (Carré et al., 2017).

The climate remarkably varies along the wide latitudinal range encompassed in the national borders due to the structural and altitudinal complexity of its territory that includes two main mountain chains: Alps (in the North) and Apennines (in Central and South). Accordingly, climate covers a broad spectrum of types ranging from polar cold and glacial (Köppen climate classification ET and EF) to Mediterranean (Cs) (Peel et al., 2007). Precipitation is moderate in the range of 350–3,500 mm per year, typically with a peak in spring and autumn, and two relative minima in winter and in summer. Italy falls into the temperate region, with a mean long-term annual air temperature of 12.6°C, spanning from ∼0°C on the Alps to around 20°C in Sicily. Additionally, a distinct continental character typifies Italy, especially the northern sectors with differences between summer and winter more than 17°C (Costantini et al., 2013).

### Aquatic Plant Data

Historical Italian floristic records (Pignatti, 1982) was compared with those reported by Conti et al. (2005, 2006), updated with the data by the “Notulae to the Italian native Vascular Flora” (NINVF) for the 2005–2015 period [from number 37(1) to number 47(1)], and with the data by the “Acta Plantarum notes” (APN) for the 2013–2015 period (from number 1 to 3). The NINVF were published by the *Informatore Botanico Italiano*, whereas the APN were published online by the “*Acta Plantarum forum.*”^1^

The historical (up to ∼2000) and the updated regional plant databases (for the period 2005–2015) ware explored in order to retrieve the number of aquatic species based on: (1) the biological form as reported by Pignatti (1982), focusing on “hydrophytes” sensu Raunkiær (1934); (2) the Ellenberg ecological indicator for “humidity” (*U*), as reported by Pignatti et al. (2005) and Guarino et al. (2012); and (3) the preferential colonized habitats, as indicated by Pignatti (1982). According to Baattrup-Pedersen et al. (2005) all species with an Ellenberg’s humidity value *U* ≥ 10 have been considered for the present analysis, being ecologically strictly related to “aquatic” habitats with permanently saturated substrates and therefore influenced by periodical submersion and/or by constant saturation of colonized sediments. Specifically, a *U* value of 10 refers to plant species adapted to transient submersion, 11 to aquatic plants rooted in waterlogged sediments but with emergent or floating organs, and 12 to submerged plants, constantly or at least for long periods (Pignatti et al., 2005). Additionally, few species with Ellenberg indicator values *U* = 9 were also considered. These species (e.g., *Montia*, *Elatine*, and *Juncus* genera and the *Veronica anagallis-aquatica* aggregate), although closely connected to aquatic ecosystems – have been classified in the past into non-aquatic life forms, or considered not obligatorily adapted to saturated sediments. Currently, new data on their ecology have permit to confirming their “aquatic life strategy.”

All the plant occurrence data were summarized into a matrix reporting the presence of each species/taxon within each region (Supplementary Table 1). Plant species were classified as follows: (1) species “no longer recorded at the local scale” since 2000 (lost; 0); (2) “dubious,” species whose confirmation requires further evaluation (?); (3) species “erroneously reported in the past,” based on exsiccata material re-examination and/or field surveys (-); and (4) species recently confirmed (+).

### Environmental Drivers

To investigate the regional arrangement of aquatic plants, a series of environmental drivers were used as explanatory variables, focusing on climatic conditions (both rainfall and temperature) and the availability of preferential habitats (**Table 1**). These data were summarized at the regional scale, the same used for assembly the plant occurrence data. Climatic variables were derived from the temperature and precipitation data sets of the ISTAT and CREA (Agricultural Mechanic Experimental Institute) 2000–2010 monthly climate time-series. This interval was used as climate reference period because it is the only period for which we have methodologically comparable and accessible data at the regional scale. Furthermore, we believe that it well describes the climatic conditions in which the floristic data in analysis were collected (2005–2015), considering a minimum/reasonable delay between the records collection and their publication of about 3–5 years.

**Table 1:** Explanatory variables used to analyze the representativeness and spatial distribution of aquatic plants in the 20 Italian regions.

Variable	Explanation	Unit	Mean	Min	Max
**Geographic**					
Longitude (*x*)					
Latitude (*y*)					
*Area*	Area of a given region	km^2^	15,066	3266	25,707
***Aquatic habitats availability***					
*Lake*	Area occupied by lentic waters by CLC data	km^2^	164.4 ^∗^ 10^6^	4.5 ^∗^ 10^6^	759.3 ^∗^ 10^6^
*Rive*	Linear development of natural hydrosystems	km	7.7 ^∗^ 10^3^	1.6 ^∗^ 10^3^	15.1 ^∗^ 10^3^
**Climate**					
*Rain*	Mean annual precipitation	mm	785	494	1077
*Aqre*	Aquifer recharge rate	km^3^ year^-1^	2754	542	5520
*Revt*	Mean annual real evapotranspiration	mm	861	601	1135
*Temp*	Mean annual temperature	°C	12.7	3.6	18.1
**Landscape**					
*Altr*	Altitude range	m	2734	1151	4537
*H*	Heterogeneity (Chappuis et al., 2012)		4.7	4.0	6.0
*Hidr*	Hydro-ecoregions heterogeneity		3.1	1.0	7.0
*Inkm*	Inhabitants per km^2^		184.4	39.0	429.0

Actual evapotranspiration was indirectly calculated based on the monthly hydrological budget model proposed by Thornthwaite–Mather (Mather, 1979); whereas, the aquifer recharge rate was estimated by the basic outflow produced by waterways integrated with the direct supply of groundwater in agreement with Castany (1982). The abundance of available aquatic habitats for the establishment and growth of aquatic plants was estimated: (1) by the total surface occupied by lakes – based on the CORINE Land Cover data^2^ (*Lake*, km^2^) – and (2) by the total linear development of natural hydrosystems – based on GIS data provided by SINAnet^3^ (*Rive*, km).

In agreement with Chappuis et al. (2012), we considered the following explanatory variables: latitude (hereafter, *y*) and longitude (*x*) (*y* and *x* coordinates of the centroid of each region); area (*Area*, km^2^); *Lake* (km^2^); *Rive* (km); mean annual precipitation (*Rain*, mm); aquifer recharge rate (*Aqre*, km^3^ year^-1^); mean annual temperature (*Temp*, °C); mean annual real evapotranspiration (*Revt*, mm); altitude range (*Altr*, m). Additionally, we considered the heterogeneity index as proposed by Chappuis et al. (2012; *H*), and a hydro-ecoregion heterogeneity index (*Hidr*) calculated as the sum of hydro-ecoregions presented at regional scale in agreement with Wasson et al. (2002). Finally, as proxy of the potential exposure rate to human perturbations we considered the population of the region (*Inkm*, inhabitants per km^2^ for 2015; ISPRA data).

### Statistical Analyses

For testing species richness patterns (updated for the period 2005–2015), we calculated three dependent variables per each region: species richness of all aquatic plants (*S_AP_*), species richness of hydrophytes (*S_HY_*), and species richness of not-obligate aquatic plants (*S_NO_*). Additionally, we also calculated the species richness of lost (*S_LO_*), dubious (*S_DU_*) and erroneously reported species (*S_ER_*) to investigate their dependence on principal environmental drivers.

The co-variation among environmental drivers was tested by the spearman rank correlation prior to data analysis and those selected were used as covariates in a generalized linear modeling framework. The collinearity levels between our predictors were relatively high (Supplementary Table 2). *X* and *y* had a significant correlation with climatic variables, especially *Revt* (*r* = 0.63, and *r* = -0.95, respectively) and *Temp* (*r* = 0.61, and *r* = -0.93, respectively). Area was strictly correlated with water resources proxies (*Lake* and *Rive*; *r* = 0.66, and *r* = 0.89, respectively); whereas, *Rain* was strictly correlated with *Temp* (*r* = -0.82). Similarly, *Revt* and *Altr* had a significant correlation with *Temp* (*r* = 0.97, and *r* = -0.69, respectively), while, *Aqre* and *Hidr* were strictly correlated with *InKm* (*r* = 0.43, and *r* = 0.62, respectively). Based on these results, we excluded *x*, *y*, *Rain*, *Aqre*, *Revt*, *Altr*, and *Hidr* from variables included in the GLM analyses devoted to detect the better predictor of aquatic plant richness.

Non-metric multidimensional scaling (NMDS) was used to study the community structure of all aquatic plants, hydrophytes and not-obligate aquatic plants among the investigated regions, with Jaccard as the dissimilarity index and stress as the measure of goodness of fit, according to Oksanen et al. (2017). Vectors of environmental variables were fitted onto the ordination obtained by NMDS and the squared correlation coefficient (*r*^2^) calculated in order to assess the goodness of fit.

Since dependent variables were discrete, we used the Poisson family for the error distribution and logarithm as link function. In order to select the best model, a multi-model inference approach was used (Burnham and Anderson, 2002). The fit of all candidate models was thus compared using Bayesian Information Criterion (BIC) and the model with the lowest BIC was retained. Correlation analysis was also performed to verify specific relations between species richness variables and environmental drivers.

All analyses were performed with the R statistical software (R Core Team, 2017), and the packages *bestglm* (Oksanen et al., 2017) and *vegan* (McLeod and Xu, 2017).

## Results

### Aquatic Plant Diversity, Distribution, and Trends

A total of 279 aquatic plant species was recognized as present in Italy in the period 2005–2015. Ten species are to be considered extinct in the wild [among which *Aldrovanda vesiculosa* L., *Caldesia parnassifolia* (Bassi ex L.) Parl., *Helosciadium repens* (Jacq.) W.J.D. Koch, and *Pilularia globulifera* L.], four were doubt [among which *Luronium natans* (L.) Raf., *Utricularia intermedia* Hayne, and *U. ochroleuca* R.W. Hartman.], and eight were erroneously reported in the past, among which *Isoëtes lacustris* L., *Myosotis rehsteineri* Wartm., and *Ranunculus aquatilis* L. (**Table 2**). Focusing on the contribution of obligate aquatic plants, the 56.5% of the total diversity (158 species) is represented by hydrophytes, whereas 43.5% (121 species) were not-obligate aquatic species. These included: 65 geophytes [equal to the 53.7%; among which *Berula erecta* (Huds.) Coville, *Cladium mariscus* (L.) Pohl, *Eleocharis palustris* (L.) Roem. & Schult. subsp. *palustris*; *Typha latifolia* L.], 33 hemicryptophytes (27.3%; among which *Nasturtium officinale* R. Br. subsp. *officinale*; *Veronica anagallis-aquatica* L., *V. beccabunga* L.), 12 therophytes [9.9%; *Montia fontana* L. subsp. *chondrosperma* (Fenzel) Walters], ten helophytes (8.3%; *Carex riparia* Curtis), and one chamaephyte [0.8%; *Potentilla palustris* (L.) Scop.].

**Table 2:** List of the lost (0), dubious (?), and erroneously reported in the past (–) aquatic species since 2000 in Italy (IT, Italy, general evaluation), and their regional distribution (VDA, Valle d’Aosta; PIE, Piedmont; LOM, Lombardy; TAA, Trentino-Alto Adige/Südtirol; VEN, Veneto; FVG, Friuli Venezia Giulia; LIG, Liguria; EMR, Emilia-Romagna; TOS, Tuscany; UMB, Umbria; MAR, Marche; LAZ, Latium; ABR, Abruzzo; MOL, Molise; CAM, Campania; PUG, Apulia; BAS, Basilicata; CAL, Calabria; SIC, Sicily; SAR, Sardinia).

Species	IT	VDA	PIE	LOM	TAA	VEN	FVG	LIG	EMR	TOS	UMB	MAR	LAZ	ABR	MOL	CAM	PUG	BAS	CAL	SIC	SAR
*Aldrovanda vesiculosa* L.	**0**	–	0	0	0	0			0	0			0				0	0			
*Azolla caroliniana* Willd.	**–**		–	–	–	–			–	–	–	–	–			–	–	–	–	–	–
*Blyxa japonica* (Miq.) Maxim. ex Asch. & Grke	**?**		0	0																	
*Caldesia parnassifolia* (Bassi ex L.) Parl.	**0**		0	0	0	?			0	0	?										
*Callitriche cribrosa* Schotsman	**0**												0								
*Cyperus flavidus* Retz.	**0**							0													
*Cyperus papyrus* L. subsp. *siculus* (Parl.) Chiov.	**–**																			–	
*Helosciadiumrepens* (Jacq.) W.J.D. Koch	**0**			0	0				0					0							
*Isoëtes lacustris* L.	**–**		–	–																	
*Isolepis fluitans* (L.) R. Br.	**0**		0				0			0											
*Luronium natans* (L.) Raf.	**?**					?															
*Myosotis rehsteineri* Wartm.	**–**		–	–																	
*Pilularia globulifera* L.	**0**		?	0		–	0					0	?	?			–				
*Ranunculus aquatilis* L.	**–**	–	–	–	–	–	–	–	–	–	–	–	–	–	–	–	–	–	–	–	–
*Ranunculus isthmicus* Boiss.	**?**																			?	
*Sagittaria platyphylla* (Engelm.) J.G. Sm.	**0**			0																	
*Stratiotes aloides* L.	**0**			0		0			0												
*Typha angustifolia* L. subsp. angustifolia	**–**	–	–	–	–	–	–	–	–	–	–	–	–	–	–	–	–	–	–	–	–
*Typha angustifolia* L. subsp. *australis* (Schum. Et Thonn.) Graebner	**–**																				
*Utricularia intermedia* Hayne	**?**			0	?	0															
*Utricularia ochroleuca* R.W. Hartman.	**?**				?																
*Zostera angustifolia* (Hornem.) Rchb.	**–**																				

Regarding the distribution of species, 18 species were present in all the regions, but only four of them are hydrophytes (*Glyceria notata* Chevall., *Lemna minor* L., *Potamogeon crispus* L., and *P. natans* L.). 19 different species were recorded at least in the 90–95% of the investigated regions (18 and 19 regions over 20), of which seven hydrophytes: *Alisma lanceolatum* With., *Callitriche stagnalis* Scop., *Lemna gibba* L., *Myriophyllum spicatum* L., *M. verticillatum* L., *Potamogeton pectinatus* L. (=*Stuckenia pectinata* (L.) Börner), and *Ranunculus trichophyllus* Chaix subsp. *trichophyllus*.

Many species had a much more restricted distribution, with 32 species being limited to a only one single region (unique species), with 17 of them being hydrophytes (as *Isoëtes sabatina* Troia & Azzella, a recently described species from Lake Bracciano, Lazio region), and 15 being not-obligate aquatic species (as *Eleocharis mamillata* H.Lindb. subsp. *mamillata*, and *Schoenoplectus carinatus* (Sm.) Palla from Friuli Venezia Giulia and Veneto regions).

**FIGURE 1:**
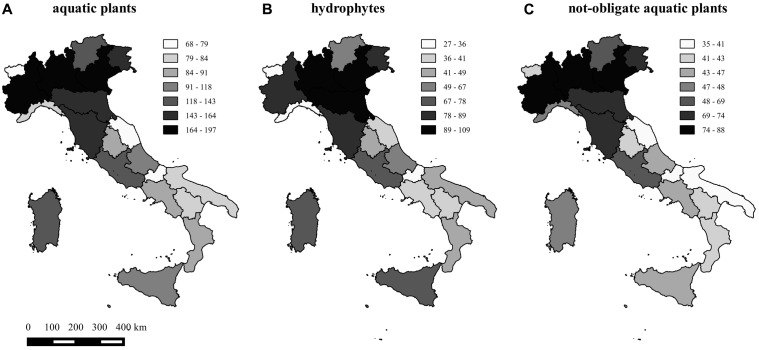
Map of the aquatic plant richness, considering the total number of species **(A)**, the total number of hydrophytes **(B)**, and the total number of not-obligate aquatic species **(C)** in the 20 regions of Italy.

**FIGURE 2:**
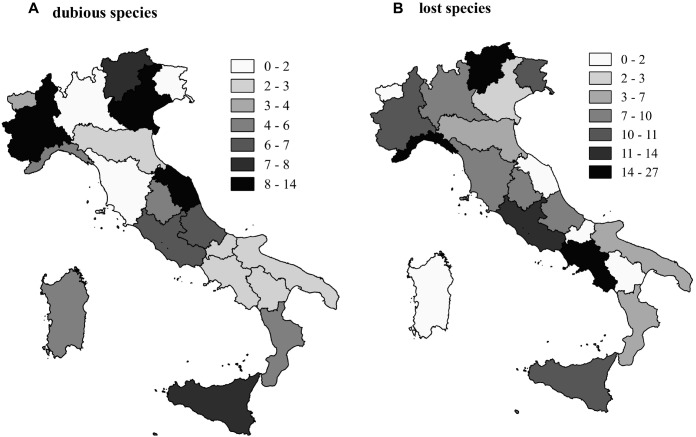
Map of the number of dubious **(A)** and lost **(B)** aquatic species since 2005 in the 20 regions of Italy.

Species richness (*S_AP_*) per region ranged from a minimum of 68 (Valle d’Aosta) to a maximum of 197 (Lombardy) and averaged 117 plants. Species richness of hydrophytes (*S_HY_*) and not-obligate aquatic species (*S_NO_*) per region ranged from 27 and 35 species up to 109 and 88, respectively, with very similar richness patterns across the 20 regions (**Figure 1**). Dubious species (*S_DU_*) ranged from 0 (Tuscany) to 14 (Piedmont), whereas “species locally no longer recorded” (*S_LO_*) ranged from 0 (Molise and Umbria regions) to 26 (Campania) (**Figure 2**).

**FIGURE 3:**
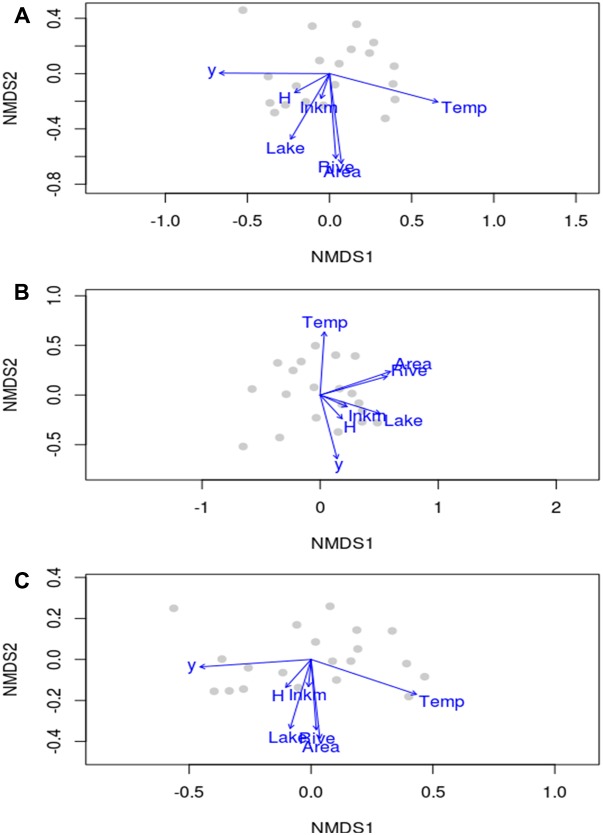
Non-metric multidimensional scaling plots of aquatic plant **(A)**, hydrophyte **(B)** and not-obligate aquatic species **(C)** composition at the regional scale in Italy, reporting the location of regions, the direction and the magnitude of environmental drivers mapped onto the ordination space. *y*, latitude; *Area*, area of a given region; *Lake*, regional total surface occupied by lakes; *Rive*, the total regional linear development of natural hydrosystems; *Temp*, regional mean annual temperature; *H*, regional heterogeneity index by Chappuis et al. (2012); *Inkm*, regional inhabitants per km^2^.

In terms of species composition, the scatterplots of aquatic plant and not-obligate aquatic species overlapped considerably in the NMDS ordination (**Figures 3A,C**). In contrast, hydrophyte plot exhibited a peculiar ordination with a lesser separation among regions (**Figure 3B**). In any case, it was not possible to identify a zonation between regions with clear differentiated clusters as an effect of rather similar aquatic plant assemblages.

### Aquatic Plant Drivers

*Temp* (i.e., mean temperature values), latitude (*y*) and *Area* were the most important drivers of regional species compositional dissimilarity by NMDS, both for the total species richness of aquatic plants, and those of hydrophytes, and not-obligate aquatic species (**Table 3**). Similarly, *Lake* (i.e., available lentic habitats for colonization) and *Rive* (i.e., available lotic habitats for colonization) exhibited *R^2^* values ≥ 0.49, suggesting a pivotal role in driving the spatial arrangement of aquatic plants across Italy (**Table 3**). On the contrary, *H* and *Inkm* did not statistically affect the regional distribution of the overall aquatic plants, and of hydrophytes and non-obligate aquatic species separately (**Table 3**). A significant dependence on the number of inhabitants per km^2^ as a proxy of local human perturbations was found exclusively for the “species locally no longer recorded” (*r* = 0.64, *p* = 0.002). Conversely, *H* seemed to have a very little influence on the total aquatic plant richness, but also considering the “species locally no longer recorded” (*r* = 0.26, *p* = 0.271), the dubious (*r* = 0.29, *p* = 0.208), and the erroneously reported species (*r* = 0.01, *p* = 0.967).

**Table 3:** Relationships between aquatic plant (*S_AP_*), hydrophyte (*S_HY_*), and not-obligate aquatic plant (*S_NO_*) regional composition (as represented in the two-dimensional NMDS space) and the following environmental drivers using ‘envfit’ in the R package ‘vegan: *y* (latitude), *Area* (area of a given regions), *Lake* (total surface occupied by lakes at the regional scale), *Rive* (the linear development of natural hydrosystems at the regional scale), *Temp* (regional mean annual temperature), *H* (regional heterogeneity index by Chappuis et al., 2012), *Inkm* (regional inhabitants per km^2^).

	*S_AP_*		*S_HY_*		*S_NO_*	
Driver	*R^2^*	*P*	*R^2^*	*P*	*R^2^*	*P*
Temp	0.88	0.001	0.71	0.001	0.89	0.001
*y*	0.83	0.001	0.76	0.001	0.87	0.001
Area	0.79	0.001	0.72	0.001	0.62	0.002
Rive	0.70	0.001	0.63	0.002	0.49	0.008
Lake	0.52	0.002	0.51	0.006	0.50	0.002
H	0.12	0.36	0.16	0.219	0.12	0.339
Inkm	0.06	0.57	0.12	0.354	0.07	0.529

Based on the GLMs, we confirmed the results obtained by NMDS emphasizing the strong effect of *Lake*, *Rive* and *Temp* on aquatic plant diversity (**Table 4**). From the best models, the regional arrangement of overall aquatic plant, hydrophyte and not-obligate aquatic species diversity related positively with *Lake* and *Rive*. Additionally, *Temp* showed a significant positive relationship with overall aquatic plant and not-obligate aquatic diversity (**Table 4**).

**Table 4:** GLM results between regional aquatic plant diversity – considering the total aquatic plant diversity (*S_AP_*), and the hydrophytic (*S_HY_*), and the not-obligate aquatic plants (*S_NO_*) diversity separately – and environmental drivers. BIC = Bayesian information criterion (means), *Lake*, regional total surface occupied by lakes; *Rive*, the total regional linear development of natural hydrosystems; and *Temp*, regional mean annual temperature. In bold the significant drivers.

Plant diversity	Best models	BIC
*S_AP_*	**8.22ˆ10*Lake*** + **4.28ˆ5*Rive*** – **0.03*Temp*** + 4.58	190.2
*S_HY_*	**1.04ˆ9*Lake*** + **4.85ˆ5*Rive*** + 3.52	167.2
*S_N__O_*	**6.74ˆ10*Lake*** + **3.24ˆ5*Rive*** – **3.53ˆ2*Temp*** + 4.07	142.8

## Discussion

### Aquatic Plant Diversity, Distribution, and Trends

The main findings of the present work confirm the key contribution of Italy to regional and global aquatic plant diversity, hosting a prevalent share (equal to the 88.5%) of the species richness present in European and Mediterranean areas, estimated at 314 different species (Chappuis et al., 2012). The species richness here check-listed also demonstrated that Italy hosts approximately 55.9% of the overall species richness of aquatic plants of the Palearctic region, and more than the 10% of the global diversity (Chambers et al., 2008). This calls for an urgent rational strategy (both at national and regional scale) to preserve aquatic plant diversity, shedding new light on the potentially dramatic consequences of local extinctions on higher spatial scales.

The high number of species recognized is due to the great variety of eco-regions and hydro-ecoregions sensu Wasson et al. (2002) present in Italy, able to guarantee optimal conditions for the establishment and growth of aquatic plants. Furthermore, Italy is also characterized by the presence of multiple climatic and edaphic conditions – from alpine to strictly Mediterranean – that exponentially increase the range of potential habitats for the life of plants. In addition, historical climate dynamics – coupled with habitat and landscape complexity – may be called into question to account for the observed aquatic plant richness rates (Nieto Feliner, 2011; Chappuis et al., 2012). Similarly, Alahuhta et al. (2013) had put emphasis on the potential “refugium” role-played by lakes occurred in supra-aquatic areas during the last Glaciation period in Finland in contributing to the current aquatic plant diversity. However, further evidences are needed to confirm the contribution of the historical aquatic plant distributions to the current ones considering their efficient dispersal mechanisms (Santamaria, 2002), as confirmed by their typical wide distributional ranges (Chambers et al., 2008).

Bilz et al. (2011) obtained very similar results to ours, indicating 270 aquatic species for Italy. However, these results are poorly comparable, considering the great methodological differences between these two studies. Hence, the key question – as stressed by Bilz et al. (2011) – is how to overcome the difficulties related to a proper selection of plants adapted to aquatic life. To do this, we decided to follow the ecological approach by Ellenberg, based on the plant “humidity” indicator values (Baattrup-Pedersen et al., 2005; Pignatti et al., 2005; Guarino et al., 2012). In this way, we have excluded several species that can be considered “tolerant” to submersion or to “saturated sediments” – as well as species of the genera *Agrostis*, *Arundo*, *Lysimachia*, and *Lythrum*, or *Mentha pulegium* L. – but which, in fact, do not have a predominant aquatic life form. It follows that the species number we obtained (279) can be considered far greater (in a certain sense, more reliable) than that reported by Bilz et al. (2011), even though this reasoning requires validation in future investigations, both at European and global scale.

The highest values of aquatic species richness have been recorded for the northern regions (Lombardy, 197; Veneto, 186; and Piedmont, 168), in line with the evidences found by Bolpagni et al. (2013) for the lowland wetlands of Lombardy. However, intermediate values of aquatic species richness were also recorded for the two major Italian islands, Sicily (116) and Sardinia (119), suggesting a non-negligible role played by local water resources availability in close relation to climatic variables (i.e., rather high mean annual temperatures). This is in agreement with several previous works that verified the contribution of both local (i.e., habitat heterogeneity), and regional (i.e., climate) drivers to macrophyte community composition in lakes (Alahuhta, 2015 and references therein).

We confirmed the high species richness of plants that can be considered “extinct in the wild” at the national scale, reinforcing the evidences of the critical status of conservation of Italian aquatic ecosystems, especially in lowlands (Bolpagni and Piotti, 2015, 2016). At regional scale, the number of lost species was well explained by the density of inhabitants, a good proxy for human disturbance. It is generally acknowledged that aquatic plants, despite represent a small fraction of the total vascular plant diversity (∼1%), are one of the most critical groups of threatened species worldwide (Saunders et al., 2002; Chambers et al., 2008). This is especially true for aquatic plants adapted e/o restricted to low altitudes, where human pressure on aquatic ecosystems is more intense (Alahuhta et al., 2013). Hence, the most impacted aquatic ecosystems are largely located at low altitudes, even in coastal sectors and along valley bottoms (below 500 m a.s.l), and show very poor water quality, as synthetized by Carré et al. (2017). They present turbid waters and an excess in nutrient availability, which favor the dominance of micro- and macroalgae, including cyanobacteria (Bolpagni et al., 2017b; Bresciani et al., 2017).

Several species were classified as “dubious” with huge variation at the regional scale (from 0 in Tuscany to 14 in Piedmont), suggesting that the present knowledge is still incomplete and there is need to fill it by future field surveys. A number of technical and practical limitations affect the effectiveness of survey campaigns in water ecosystems compared to terrestrial ones, justifying, in part, the current lack of updated information on these systems (Azzella et al., 2013). Despite this, at national level a remarkable revival of interest in aquatic flora was stimulated by the enactment of the WFD (Bolpagni et al., 2017a). It has resulted into a renewed attention for inland aquatic habitats in general, and it has favored the integration of the available aquatic plant knowledge (Testi et al., 2009; Ceschin et al., 2010; Azzella et al., 2013, 2014; Villa et al., 2015; Abati et al., 2016; Bolpagni et al., 2016). Nevertheless, much work has to be done in order to facilitate the comparison and sharing of information gathered by the various institutional actors involved in the monitoring programs.

A non-negligible number of species “erroneously reported in the past” was also recognized. In general, these species that are quite difficult to be properly identified, due to the extreme “lability” of the morphological characters used for classification, or because they could be considered cryptic species. This may explain why these entities – recognized in the past – have been not recently confirmed. Examples in this sense are given by *M. rehsteineri*, *Ranunculus aquatilis* L., and *Isoetes lacustris* L. *M. rehsteineri* is a very rare species that is very similar to the congeneric *Myosotis scorpioides* L., to which should be assigned the Italian records of *M. rehsteineri, before* 1980s. *R. aquatilis* was detailed investigated by Desfayes (2008) and, on the basis of comprehensive collections from North Italy and Sardinia, this author suggested that this species must be excluded from the Italian flora, and its historical records should largely be assigned to *Ranunculus penicillatus* (Dumort.) Bab. s.l. or *R. peltatus* Schrank s.l. (Desfayes, 2011). *I. lacustris* was erroneously indicated for specimens collected in the Lake Orta in the mid-19th century by De Notaris (1848), which actually are to be reported to *Isoetes echinospora* Durieu, as well as all the subsequent records of the species at the national scale (Troia and Greuter, 2015).

### Ecological Drivers of Aquatic Plant Diversity

The relative availability of water resources emerged as a major driver in explaining the high level of species diversity of aquatic plants observed at regional scale. In fact, the regional surface occupied by lakes and the regional length of natural hydrosystems were found to be the predominant factors in the GLM models predicting aquatic species richness (**Table 4**). It may seem obvious, considering the strictly dependence of aquatic plants to water (see Chappuis et al., 2012). Even though, in none of the models rainfall and other climate variables – with the exception of mean annual temperature – were statistically significant. Chappuis et al. (2012) collected similar evidences, highlighting a strong interplay between temperature and water availability, in turn intimately interconnected with latitude. This is probably due to the peculiar geographic structure of the Italian peninsula, strictly oriented along the latitudinal North–South gradient, and characterized by the presence of significant mountain chains in almost all the Italian regions. The lowest altitude range recorded was equal to 1151 m, with a mean value of 2734 m (**Table 1**), suggesting the presence of a complex mosaic of habitats (i.e., high level of heterogeneity) within each considered region, that is a relatively narrow geographic context. Hence, the observations by Alahuhta et al. (2017) verified the pivotal contribution of environmental heterogeneity in driving the global pattern of macrophyte species richness among lakes, stressing on the role of climate-related restrictions (altitudinal-grown limitation) and water quality gradients (at low altitudes). Despite this, our results tended to minimize the role of heterogeneity on aquatic plant arrangement. This is probably due to the high rate of shared heterogeneity among regions, masking its contribution to the observed patterns of plant diversity.

Based on the present data, the regional patterns of aquatic plant in Italy seem, in the small, to mirror the general spatial models elaborated to explain aquatic plant distribution at larger scales. A quite clear geographical trend was observed: moving from the North to the South of the peninsula a progressive reduction in aquatic plant diversity was noted. This complements the findings by Chappuis et al. (2012), which verified a peak of aquatic plant diversity around 50° N. Additionally, we confirmed the tight overlap between the predictable latitudinal trend and the climate gradients (i.e., temperature and precipitation). Similarly, the water resources (*Rive* and *Lake*) were intimately linked to region’s area (**Figure 3** and **Table 3**). Hence, as generally expected the aquatic plant richness was strictly positively related to the area investigated, and to the sampling effort carried out (Chappuis et al., 2012; Alahuhta et al., 2013).

### Implications for Global Aquatic Plants Conservation in a Changing World: Suggestions from the Italian Case

The findings of this survey confirm the combined effects of climate change and the direct human impacts on aquatic ecosystems as the leading driver for the long-term conservation of aquatic plants both at regional and global scale (Li et al., 2006; Chambers et al., 2008). Based on historical and current evidences, Italy can play a non-negligible role in guarantee medium to high levels of species diversity for aquatic plans at multiple scales, acting as temporary refuge for projected or expected species migrations along the latitude and longitude gradients. Future scenarios for Italy suggest large changes in precipitation patterns and temperatures with huge effects on river discharges (Billi and Fazzini, 2017). This is expected, especially, for the Alpine and pre-Alpine sectors in the northern Italian regions (Coppola and Giorgi, 2010; Marchina et al., 2017), that host the prevalent share of the national aquatic plant diversity. Focusing on the Po plain, mid-term (average increases, with a Representative Concentration Pathway of 4.5, at 2050, mean forecast) predictive models suggest a clear increase in temperature descriptors. This is true, for example, in terms of the “highest temperature in the warmer month” that should range from 29.3 (current conditions) to 31.7–32.4°C range (depending on the forecast model chosen, CNRM-CM5 or MPI-ESM-LR, respectively; WorldClim 2 dataset; Fick and Hijmans, 2017).

In the short and mid-terms, these predictions imply worse conditions for aquatic plants, suggesting the need of urgent “conservation actions” to lower the current loss of aquatic plant diversity. In this context, all the aquatic ecosystems presented in a specific area, including the man-made water bodies, must be considered crucial for plants conservation overcoming classical paradigms in biodiversity conservation. In other words, the production system (mainly farming and agriculture, but also recreational areas in urban settlements) must play a central role in taking care the diversity of aquatic ecosystems considering its advantages in terms of products value, as well as functional services rendered to human. For example, the artificial network of ditches and channels in agricultural areas – especially if intensive – could act as a temporary refuge for rare and threatened plant species (Bolpagni et al., 2013; Bolpagni and Piotti, 2016). Similarly, artificial basins such as quarry lakes or irrigation reservoirs along river courses can mimic the alternating phases of formation and destruction of marginal aquatic environments counteracting the loss of fluvial dynamics. Accordingly, the use of more proper management practices for the secondary hydrographic network and artificial water bodies is a key strategy to both guarantee local plant diversity and improve more rapid and effective adaptive responses to future critical conditions. Focusing on lowlands, a strategic option is to valorise and support organic farming systems, which has largely proven to be winning in reducing energy consumption, local pollution (especially at ground- and superficial water level) and supporting diversity (Dalzochio et al., 2016; Martínez-Eixarch et al., 2017). In addition, it is also essential to elaborate lasting strategies able to counteract effectively the future critical conditions. To do this, a better integration among disciplines would be desirable, for example reinforcing the relations between legislative policies devoted to valorise natural resources. In Europe, a paradigmatic example of such a situation is the potential synergies between the WFD and the Habitats Directive in the management of water bodies, as well as aquatic habitats and biodiversity (Ecke et al., 2010; Bolpagni et al., 2017a).

A better comprehension of the contribution of aquatic plants to habitats functioning is equally fundamental, considering the key processes regulated by primary producers in water as sediment re-oxygenation, C and nutrient cyclization, sediment stabilization, algal bloom control, etc. (Chambers et al., 2008; Soana and Bartoli, 2014; O’Hare et al., 2017). This can support a new awareness on the importance of aquatic plants in high-stressed areas such as lowlands or wetland contexts where frequently aquatic ecosystems do not meet minimum quality standards. Hence, the maintenance of submerged plant meadows in drainage channels can improve the capability of semi-natural secondary hydrographic network to control N and P availability with enormous benefits for both natural and human uses (Castaldelli et al., 2015; Soana et al., 2017). Similarly, a plenty of studies have verified the pivotal role of hydrophytes in controlling the oxygen and C balances in stagnant waters (Bolpagni et al., 2007; Pierobon et al., 2010).

Based on these evidences, we are quite convinced that the ability to rise up the awareness by the stakeholders and the general public is essential to break down the slow but inexorable loss of aquatic plants. All that is strictly related to our ability to emphasize the crucial contribution of aquatic ecosystems (and hydrophytes) to local and global economy and human well-being.

## Author Contributions

RB designed the research, contributed and coordinated the assembly of the Italian aquatic plant database. CS contributed to assembly the database, and AL processed and analyzed the data with help from RB. RB coordinated the writing of the manuscript, with contribution by all authors, especially by AC, who is the project coordinator of this work.

## Conflict of Interest Statement

The authors declare that the research was conducted in the absence of any commercial or financial relationships that could be construed as a potential conflict of interest.

## Footnotes

1 http://www.actaplantarum.org/flora/flora.php

2 https://www.eea.europa.eu/data-and-maps/

3 http://www.sinanet.isprambiente.it/it
