# Supplementary material for: Aquatic Plant Diversity in Italy: Distribution, Drivers and Strategic Conservation Actions
Source: Front Plant Sci. 2018 Feb 13;9:116. doi: 10.3389/fpls.2018.00116 (PMC5816802; doi:10.3389/fpls.2018.00116)
Supplement: Supplementary file 1 [file Table_1.docx]

Supplementary Material

Aquatic plant diversity in Italy: regional distribution, trends and drivers

**Rossano Bolpagni*, Alex Laini, Chiara Stanzani, Alessandro Chiarucci**

*** Correspondence:** Corresponding Author: rossano.bolpagni@unipr.it

# Supplementary Figures and Tables

For more information on Supplementary Material and for details on the different file types accepted, please see [here](http://home.frontiersin.org/about/author-guidelines#SupplementaryMaterial).

## Supplementary Tables

**Supplementary Table 1.** Regional distribution of the Italian aquatic plants.

| **Species** | **VDA** | **PIE** | **LOM** | **TAA** | **VEN** | **FVG** | **LIG** | **EMR** | **TOS** | **UMB** | **MAR** | **LAZ** | **ABR** | **MOL** | **CAM** | **PUG** | **BAS** | **CAL** | **SIC** | **SAR** |
| --- | --- | --- | --- | --- | --- | --- | --- | --- | --- | --- | --- | --- | --- | --- | --- | --- | --- | --- | --- | --- |
| *Acorus calamus* L. |  |  | + |  | + |  | + | + |  |  |  |  |  |  |  |  |  |  |  |  |
| *Alisma gramineum* Lej. subsp*. gramineum* |  |  | + |  |  |  |  | + |  |  |  |  |  |  |  |  |  |  |  |  |
| *Alisma lanceolatum* With. |  | + | + | + | + | + | + | + | + | + | + | + | + | + | + | + | + |  | + | + |
| *Alisma plantago-aquatica* L. | + | + | + | + | + | + | + | + | + | + | + | + | + | + | + | + | + | + | + | + |
| *Alternanthera philoxeroides* (Mart.) Griseb. |  |  |  |  |  |  |  |  | + |  |  | + |  |  |  |  |  |  |  |  |
| *Althenia filiformis* Petit subsp. *filiformis* |  |  |  |  |  |  |  | + | + |  |  |  |  |  |  | + |  |  | + | + |
| *Arabis soyeri* Reut. & A. Huet subsp. *subcoriacea* (Gren.) Breistr. | + | + | + | + | + | + | + |  |  |  |  |  |  |  |  |  |  |  |  |  |
| *Azolla filiculoides* Lam. |  | + | + |  | + |  |  | + | + | + | + | + |  |  | + | + | + | + | + | + |
| *Baldellia ranunculoides* (L.) Parl. |  |  | + |  | + | + | + | + | + |  |  | + | + |  | + | + |  |  |  | + |
| *Berula erecta* (Huds.) Coville | + | + | + | + | + | + | + | + | + | + | + | + | + | + | + | + | + | + | + | + |
| *Bolboschoenus glaucus* (Lam.) S.G. Sm. |  |  |  |  | + |  |  | + |  |  |  | + |  |  |  |  | + |  |  |  |
| *Bolboschoenus laticarpus* Marhold. et al. |  | + | + |  | + |  |  | + |  |  |  |  |  |  |  |  |  |  |  |  |
| *Bolboschoenus maritimus* (L.) Palla |  |  | + | + | + |  |  | + | + |  |  |  |  |  |  |  | + |  |  | + |
| *Bolboschoenus planiculmis* (F. Schmidt.) T.V. Egorova |  | + | + |  | + |  |  | + |  |  |  |  |  |  |  |  |  |  |  |  |
| *Butomus umbellatus* L. |  | + | + | + | + | + |  | + | + | + |  | + | + |  |  |  |  |  |  | + |
| *Callitriche brutia* Petagna |  | + | + |  | + |  |  |  | + | + | + | + | + |  |  | + | + | + | + | + |
| *Callitriche cophocarpa* Sendtn. |  |  |  | + | + | + |  | + | + |  |  | + |  | + |  |  |  |  | + |  |
| *Callitriche hamulata* Kütz. ex W.D.J. Koch |  | + | + |  | + | + |  | + | + | + |  | + |  | + |  |  |  |  | + |  |
| *Callitriche hermaphroditica* L. |  |  |  |  |  |  |  |  | + |  |  |  |  |  |  |  |  |  |  |  |
| *Callitriche lenisulca* Clavaud |  |  | + |  | + |  |  | + |  |  |  |  |  | + |  |  |  |  | + |  |
| *Callitriche obtusangula* Le Gall |  | + | + | + | + | + |  |  | + |  |  | + | + |  |  |  |  |  | + | + |
| *Callitriche palustris* L. | + | + | + | + | + | + |  | + | + | + | + | + | + | + |  | + | + |  |  | + |
| *Callitriche platycarpa* Kütz. |  | + | + |  | + |  |  |  |  |  |  |  |  | + |  |  |  |  |  |  |
| *Callitriche regis-jubae* Schotsman |  |  |  |  |  |  |  |  | + |  |  |  |  |  |  |  |  |  |  | + |
| *Callitriche stagnalis* Scop. |  | + | + | + | + | + | + | + | + | + | + | + | + | + | + | + | + | + | + | + |
| *Callitriche truncata* Guss. subsp. *occidentalis* (Rouy) Schotsman |  |  |  |  |  |  |  |  |  |  |  |  |  |  |  |  |  |  | + |  |
| *Callitriche truncata* Guss. subsp. *truncata* |  |  |  |  |  |  |  |  |  |  |  | + |  |  |  |  |  |  | + | + |
| *Carex bicolor* All. | + | + | + | + | + |  |  |  |  |  |  |  |  |  |  |  |  |  |  |  |
| *Carex buekii* Wimm. |  |  |  |  |  | + |  |  |  |  |  |  |  |  |  |  |  |  |  |  |
| *Carex buxbaumii* Wahlenb. |  | + |  |  |  | + |  |  |  | + |  |  | + |  |  |  |  |  |  |  |
| *Carex elata* All. | + | + | + | + | + | + |  | + | + | + | + | + | + |  | + |  |  |  |  | + |
| *Carex fuliginosa* Schkuhr |  |  | + | + |  | + |  |  |  |  |  |  |  |  |  |  |  |  |  |  |
| *Carex hartmanii* Cajander |  | + |  | + | + |  |  |  |  |  |  |  |  |  |  |  |  |  |  |  |
| *Carex juncella* (Fr.) Th. Fr. |  | + |  |  |  |  |  | + |  |  |  |  |  |  |  |  |  |  |  |  |
| *Carex microglochin* Wahlenb. | + | + | + | + | + |  |  |  |  |  |  |  |  |  |  |  |  |  |  |  |
| *Carex panormitana* Guss. |  |  |  |  |  |  |  |  |  |  |  |  |  |  |  |  |  |  | + | + |
| *Carex paupercula* Michx. | + | + | + | + | + | + |  | + |  |  |  |  |  |  |  |  |  |  |  |  |
| *Carex pseudocyperus* L. |  | + | + | + | + | + |  | + | + | + |  | + | + | + | + |  | + | + |  |  |
| *Carex punctata* Gaudin | + | + | + | + | + |  | + | + | + |  | + | + | + | + | + | + | + | + | + | + |
| *Carex riparia* Curtis |  | + | + | + | + | + | + | + | + | + |  | + | + | + | + | + | + | + | + | + |
| *Carex rostrata* Stokes | + | + | + | + | + | + | + | + | + |  |  | + | + |  |  |  | + | + |  |  |
| *Ceratophyllum demersum* L. |  | + | + | + | + | + | + | + | + | + |  | + | + |  | + | + | + | + | + | + |
| *Ceratophyllum submersum* L. |  | + | + | + | + | + |  | + | + |  | + | + | + |  |  | + |  | + | + |  |
| *Cladium mariscus* (L.) Pohl | + | + | + | + | + | + | + | + | + | + | + | + | + | + | + | + | + | + | + | + |
| *Cymodocea nodosa* (Ucria) Asch. |  |  |  |  | + | + | + | + | + |  | + | + | + | + | + | + | + | + | + | + |
| *Cyperus badius* Desf. |  |  |  |  | + |  | + | + | + |  |  | + | + | + | + |  | + | + | + | + |
| *Cyperus congestus* Vahl |  | + | + |  | + |  |  |  |  |  |  |  |  |  |  |  |  |  |  |  |
| *Cyperus difformis* L. |  | + | + |  | + | + |  | + | + |  | + |  |  |  |  |  |  | + | + | + |
| *Cyperus esculentus* L. |  | + | + | + | + | + | + | + | + |  |  | + |  | + | + | + | + | + | + |  |
| *Cyperus glaber* L. |  |  | + |  | + |  |  | + |  |  |  |  |  |  |  | + | + | + | + | + |
| *Cyperus glomeratus* L. | + | + | + | + | + | + | + | + | + |  | + |  |  | + | + |  |  | + |  |  |
| *Cyperus longus* L. |  | + | + | + | + | + | + | + | + | + | + | + | + | + | + | + | + | + | + | + |
| *Cyperus papyrus* L. subsp. *papyrus* |  |  |  |  |  |  |  |  |  |  |  |  |  |  |  |  |  |  | + |  |
| *Cyperus polystachyus* Rottb. |  |  |  |  |  |  |  |  |  |  |  | + |  |  | + |  |  |  |  |  |
| *Cyperus serotinus* Rottb. |  | + | + |  | + | + | + | + | + |  |  | + |  |  |  |  |  |  |  |  |
| *Cyperus squarrosus* L. |  | + | + |  | + |  |  | + |  |  |  |  |  |  |  |  |  |  |  |  |
| *Cyperus strigosus* L. |  | + | + |  | + |  |  | + |  |  |  |  |  |  |  |  |  |  |  |  |
| *Damasonium alisma* Mill. subsp. *alisma* |  |  |  |  |  |  |  |  | + |  |  |  |  |  |  | + |  |  | + |  |
| *Damasonium alisma* Mill. subsp. *bourgaei* (Coss.) Maire |  |  |  |  |  |  |  |  |  |  |  |  |  |  |  |  | + |  | + | + |
| *Damasonium polyspermum* Coss. |  |  |  |  |  |  |  |  |  |  |  |  |  |  |  | + |  |  | + |  |
| *Egeria densa* Planch. |  | + | + |  |  |  |  | + | + |  |  |  |  |  | + |  |  |  |  |  |
| *Eichhornia crassipes* (Mart.) Solms |  |  | + |  | + | + |  | + | + |  |  | + |  |  | + |  |  |  | + | + |
| *Elatine alsinastrum* L. |  | + | + |  |  |  |  |  |  |  |  | + | + |  |  |  |  |  | + | + |
| *Elatine ambigua* Wight |  | + | + |  |  |  |  | + |  |  |  |  |  |  |  |  |  |  |  |  |
| *Elatine gussonei* (Sommier) Brullo et al. |  |  |  |  |  |  |  |  |  |  |  |  |  |  |  |  |  |  | + |  |
| *Elatine hexandra* (Lapierre) DC. |  | + | + |  | + |  |  | + |  |  |  |  |  |  |  |  |  |  |  |  |
| *Elatine hydropiper* L. |  |  | + |  |  |  |  |  |  |  |  |  |  |  |  |  |  |  |  |  |
| *Elatine macropoda* Guss. |  |  |  |  |  |  |  |  |  |  |  |  |  |  |  | + |  |  | + | + |
| *Elatine triandra* Schkuhr |  | + | + |  | + |  |  | + |  |  |  |  |  |  |  |  |  | + |  | + |
| *Eleocharis acicularis* (L.) Roem. & Schult. | + | + | + | + | + | + |  | + | + | + |  | + |  |  | + |  | + | + |  |  |
| *Eleocharis carniolica* Koch |  | + | + |  |  | + |  |  | + |  |  |  |  |  |  |  |  |  |  |  |
| *Eleocharis flavescens* (Poir.) Urb. |  | + | + |  |  |  |  |  |  |  |  |  |  |  |  |  |  |  |  |  |
| *Eleocharis geniculata* (L.) Roem. & Schult. |  |  |  |  |  |  |  |  | + |  |  |  |  |  |  |  |  |  |  | + |
| *Eleocharis mamillata* H. Lindb. subsp. *austriaca* (Hayek) Strandh. | + | + | + | + | + | + |  |  |  |  |  |  |  |  |  |  |  |  |  |  |
| *Eleocharis mamillata* H. Lindb. subsp. *mamillata* |  |  |  |  |  | + |  |  |  |  |  |  |  |  |  |  |  |  |  |  |
| *Eleocharis multicaulis* (Sm.) Desv. |  | + | + |  | + |  |  |  | + |  |  |  |  |  |  | + |  |  |  | + |
| *Eleocharis palustris* (L.) Roem. & Schult. subsp. *palustris* | + | + | + | + | + | + | + | + | + | + | + | + | + | + | + | + | + | + | + | + |
| *Eleocharis parvula* (Roem. & Schult.) Link ex Bluff, Nees & Schauer |  |  |  |  | + | + |  |  |  |  |  |  |  |  |  |  |  |  |  | + |
| *Eleocharis pellucida* J. Presl & C. Presl |  | + |  |  |  |  |  |  |  |  |  |  |  |  |  |  |  |  |  |  |
| *Eleocharis quinqueflora* (Hartmann) O. Schwarz | + | + | + | + | + | + |  | + | + | + | + | + | + |  |  |  |  | + |  |  |
| *Eleocharis uniglumis* (Link) Schult. subsp. *uniglumis* | + |  | + | + | + | + | + | + | + | + | + | + | + | + |  |  | + | + |  |  |
| *Elodea canadensis* Michx. |  | + | + | + | + | + | + | + | + | + |  | + | + | + |  |  |  |  |  |  |
| *Elodea nuttallii* (Planch.) H. St.John |  |  | + | + | + | + |  | + |  |  |  |  |  |  |  |  |  |  |  |  |
| *Equisetum fluviatile* L. | + | + | + | + | + | + | + | + | + | + | + | + | + | + | + |  | + | + |  |  |
| *Eriophorum angustifolium* Honck. | + | + | + | + | + | + | + | + | + |  |  |  |  |  |  |  |  |  |  |  |
| *Eriophorum gracile* W.D.J. Koch ex Roth |  |  |  | + | + |  |  |  | + |  |  |  |  |  |  |  |  |  |  |  |
| *Eriophorum latifolium* Hoppe | + | + | + | + | + | + | + | + | + |  | + | + | + |  |  |  |  |  |  |  |
| *Eriophorum scheuchzeri* Hoppe | + | + | + | + | + | + | + | + | + |  |  |  |  |  |  |  |  |  |  |  |
| *Eriophorum vaginatum* L. | + | + | + | + | + | + |  |  |  |  |  |  |  |  |  |  |  |  |  |  |
| *Glyceria x pedicellata* F. Towns |  |  |  | + |  |  |  |  |  |  |  |  |  |  |  |  |  |  |  |  |
| *Glyceria fluitans* (L.) R. Br. | + | + | + | + | + | + | + | + | + | + | + | + | + | + | + | + | + | + | + | + |
| *Glyceria maxima* (Hartm.) Holmb. |  | + | + | + | + | + |  | + | + | + | + |  | + | + | + |  |  |  | + | + |
| *Glyceria notata* Chevall. | + | + | + | + | + | + | + | + | + | + | + | + | + | + | + | + | + | + | + | + |
| *Groenlandia densa* (L.) Fourr. | + | + | + | + | + |  |  | + |  | + |  | + | + | + | + | + | + | + | + |  |
| *Halogeton sativus* (L.) Moq. |  |  |  |  |  | + |  |  |  |  |  |  |  |  |  |  |  |  |  |  |
| *Halophila stipulacea* (Forssk.) Asch. |  |  |  |  | + |  |  |  |  |  |  |  |  |  |  | + |  |  | + |  |
| *Helosciadium crassipes* W.J.D. Koch ex Rchb. |  |  |  |  |  |  |  |  |  |  |  | + |  |  |  |  |  |  | + | + |
| *Helosciadium inundatum* (L.) W.J.D. Koch |  |  |  |  |  |  |  |  | + |  |  | + |  | + |  |  |  |  | + |  |
| *Helosciadium nodiflorum* (L.) W.J.D. Koch |  | + | + | + | + | + | + | + | + | + | + | + | + | + | + | + | + | + | + | + |
| *Heteranthera limosa* Willd. |  | + | + |  |  |  |  | + |  |  |  |  |  |  |  |  |  |  |  | + |
| *Heteranthera reniformis* Ruiz & Pav. |  | + | + |  | + |  |  | + | + |  |  |  |  |  |  |  |  |  |  |  |
| *Heteranthera rotundifolia* (Kunth) Griseb. |  | + | + |  |  |  |  | + |  |  |  |  |  |  |  |  |  |  |  | + |
| *Hippuris vulgaris* L. |  | + | + | + | + | + |  | + | + | + |  | + |  |  | + |  |  |  |  |  |
| *Hottonia palustris* L. |  | + | + |  | + | + |  | + | + |  |  | + |  |  |  |  |  |  |  |  |
| *Hydrilla verticillata* (L.f.) Royle |  |  |  |  | + |  |  |  |  |  |  |  |  |  |  |  |  |  |  |  |
| *Hydrocharis morsus-ranae* L. |  | + | + | + | + | + | + | + | + | + |  | + |  |  |  |  |  |  |  |  |
| *Hydrocotyle ranunculoides* L. f. |  |  |  |  |  |  |  |  | + |  |  | + |  |  | + |  |  |  |  | + |
| *Hydrocotyle vulgaris* L. |  |  | + |  | + | + |  | + | + | + |  | + |  |  | + | + |  | + |  | + |
| *Hypericum elodes* L. |  |  |  |  |  |  |  |  | + |  |  |  |  |  |  |  |  |  |  |  |
| *Iris pseudacorus* L. |  | + | + | + | + | + | + | + | + | + | + | + | + | + | + | + | + | + | + | + |
| *Isoëtes duriei* Bory |  |  |  |  |  |  | + |  | + |  |  | + |  |  | + |  |  | + | + | + |
| *Isoëtes echinospora* Durieu |  | + | + |  |  |  |  |  |  |  |  |  |  |  |  |  |  |  |  |  |
| *Isoëtes gymnocarpa* (Gennari) A. Braun |  |  |  |  |  |  |  |  |  |  |  |  |  |  |  |  |  | + | + | + |
| *Isoëtes histrix* Bory |  |  |  |  |  |  |  |  | + | + |  | + |  |  |  | + |  | + | + | + |
| *Isoëtes longissima* Bory |  |  |  |  |  |  |  |  | + |  |  | + |  |  |  | + |  |  | + | + |
| *Isoëtes malinverniana* Ces. & De Not. |  | + | + |  |  |  |  |  |  |  |  |  |  |  |  |  |  |  |  |  |
| *Isoëtes sabatina* Troia & Azzella |  |  |  |  |  |  |  |  |  |  |  | + |  |  |  |  |  |  |  |  |
| *Isoëtes tigulliana* Gennari |  |  |  |  |  |  |  |  |  |  |  |  |  |  |  |  |  |  |  | + |
| *Isoëtes todaroana* Troìa & Raimondo |  |  |  |  |  |  |  |  |  |  |  |  |  |  |  |  |  | + | + |  |
| *Juncus acutiflorus* Ehrh. ex Hoffm. | + | + | + | + | + | + | + | + | + | + | + | + | + |  | + | + | + |  | + | + |
| *Juncus alpinoarticulatus* Chaix | + | + | + | + | + | + | + | + | + |  | + | + | + |  |  |  |  |  |  |  |
| *Juncus bulbosus* L. |  | + | + | + | + | + |  | + | + | + |  | + |  |  |  |  |  | + |  |  |
| *Juncus dichotomus* Elliott |  | + | + |  |  |  |  |  |  |  |  |  |  |  |  |  |  |  |  |  |
| *Juncus effusus* L. | + | + | + | + | + | + | + | + | + | + | + | + | + | + | + | + | + | + | + | + |
| *Juncus ensifolius* Wikstr. |  |  |  | + |  |  |  |  |  |  |  |  |  |  |  |  |  |  |  |  |
| *Juncus filiformis* L. | + | + | + | + | + | + | + | + | + |  |  |  | + |  |  |  |  |  |  |  |
| *Juncus fontanesii* J. Gay |  |  | + |  |  |  | + | + | + |  | + | + | + | + |  | + |  | + | + | + |
| *Juncus heterophyllus* Desf. |  |  |  |  |  |  |  | + | + |  |  | + |  |  |  |  |  | + | + | + |
| *Juncus inflexus* L. | + | + | + | + | + | + | + | + | + | + | + | + | + | + | + | + | + | + | + | + |
| *Juncus marginatus* Rostk. |  | + |  |  |  |  |  |  |  |  |  |  |  |  |  |  |  |  |  |  |
| *Juncus squarrosus* L. |  | + |  |  |  |  |  |  |  |  |  |  |  |  |  |  |  |  |  |  |
| *Juncus subnodulosus* Schrank | + | + | + | + | + | + | + | + | + | + | + | + | + | + | + | + | + | + | + | + |
| *Juncus thomasii* Ten. |  |  |  |  |  |  |  |  |  |  |  |  |  |  |  |  | + | + |  |  |
| *Juncus triglumis* L. | + | + | + | + | + | + |  |  |  |  |  |  | + |  |  |  |  |  |  |  |
| *Kyllinga brevifolia* Rottbøll |  |  | + |  |  |  |  |  |  |  |  |  |  |  |  |  |  |  |  |  |
| *Lagarosiphon major (Ridl.) Moss* |  | + | + | + | + |  |  | + |  |  |  |  |  |  |  |  |  |  |  |  |
| *Landoltia punctata* (C. Mey.) Les. & D.J. Crawford |  | + | + |  |  |  |  |  |  |  |  |  |  |  |  |  |  |  |  |  |
| *Leersia oryzoides* (L.) Sw. |  | + | + | + | + | + | + | + | + | + | + | + |  |  | + |  |  | + |  | + |
| *Lemna aequinoctialis* Welw. |  | + | + |  | + |  |  | + |  |  |  |  |  |  |  |  |  |  |  | + |
| *Lemna gibba* L. |  | + | + | + | + | + |  | + | + | + | + | + | + | + | + | + | + | + | + | + |
| *Lemna minor* L. | + | + | + | + | + | + | + | + | + | + | + | + | + | + | + | + | + | + | + | + |
| *Lemna minuta* Kunth |  | + | + | + | + | + |  | + | + | + | + | + | + |  |  | + |  | + | + | + |
| *Lemna trisulca* L. |  | + | + | + | + | + |  | + | + | + |  | + | + | + | + | + | + |  | + | + |
| *Lemna valdiviana* Phil. |  |  |  |  |  |  |  |  |  |  |  | + |  |  |  |  |  |  |  | + |
| *Limnophila x ludoviciana* Thieret |  |  |  |  | + |  |  | + |  |  |  |  |  |  |  |  |  |  |  |  |
| *Ludwigia hexapetala* (Hook. & Arn.) Zardini, H. Gu & P.H. Raven |  |  | + |  | + |  |  | + |  |  |  |  |  |  |  |  |  |  |  |  |
| *Ludwigia peploides* (Kunth) P.H. Raven subsp. *montevidensis* (Spreng.) P.H. Raven |  | + | + |  | + |  |  | + | + |  |  | + |  |  |  |  |  |  |  |  |
| *Marsilea quadrifolia* L. |  | + | + | + | + |  |  | + | + |  |  |  |  |  |  |  |  |  |  |  |
| *Marsilea strigosa* Willd. |  |  |  |  |  |  |  |  |  |  |  |  |  |  |  | + | + |  |  | + |
| *Menyanthes trifoliata* L. | + | + | + | + | + | + | + | + | + | + |  | + | + | + | + |  |  | + |  |  |
| *Monochoria korsakowii* Regel & Maack |  |  | + |  |  |  |  |  |  |  |  |  |  |  |  |  |  |  |  |  |
| *Montia fontana* L. subsp. *amporitana* Sennen |  |  | + |  |  |  |  |  | + |  |  |  |  |  |  |  |  |  | + | + |
| *Montia fontana* L. subsp. *chondrosperma* (Fenzel) Walters |  | + | + | + | + |  |  | + | + | + | + | + | + | + | + |  | + | + | + | + |
| *Montia fontana* L. subsp. *fontana* |  | + | + | + | + |  |  |  |  |  |  |  |  |  |  |  |  |  |  |  |
| *Montia fontana* L. subsp. *variabilis* Walters |  |  |  | + |  |  |  |  |  |  |  |  |  |  |  |  |  |  | + | + |
| *Myosotis laxa* Lehm. |  | + | + | + | + | + | + |  | + | + |  | + | + | + | + |  |  |  |  |  |
| *Myosotis scorpioides* L. | + | + | + | + | + | + | + | + | + | + | + | + | + | + | + |  | + |  |  |  |
| *Myriophyllum alterniflorum* DC. |  |  | + |  |  |  |  |  | + |  |  | + |  |  |  |  |  | + | + | + |
| *Myriophyllum aquaticum* (Velloso) Verdc. |  | + | + |  | + | + |  |  | + |  | + | + |  |  | + |  |  |  |  |  |
| *Myriophyllum spicatum* L. |  | + | + | + | + | + | + | + | + | + | + | + | + |  | + | + | + | + | + | + |
| *Myriophyllum verticillatum* L. | + | + | + | + | + | + | + | + | + | + | + | + | + | + | + | + | + |  | + |  |
| *Najas gracillima* (A. Braun) Magnus |  | + | + |  | + |  |  | + |  |  |  |  |  |  |  |  |  | + |  |  |
| *Najas graminea* Delile |  |  | + |  |  |  |  | + |  |  |  |  |  |  |  |  |  |  |  |  |
| *Najas marina* L. subsp. *armata* (H. Lindb.) Horn |  |  |  |  |  |  |  |  |  |  |  |  |  |  |  |  |  |  |  | + |
| *Najas marina* L. subsp. *marina* |  | + | + | + | + | + |  | + | + | + | + | + |  |  | + |  |  |  |  | + |
| *Najas minor* All. |  | + | + | + | + | + | + | + | + | + |  | + |  |  |  |  |  |  |  |  |
| *Nanozostera noltii* (Hornem.) Toml. & Posl. |  |  |  |  | + | + |  | + | + |  | + | + | + | + | + | + | + | + | + | + |
| *Nasturtium microphyllum* Boenn. ex Rchb. |  | + | + | + | + | + | + |  |  |  |  |  |  |  |  |  |  |  |  |  |
| *Nasturtium officinale* R. Br. subsp. *officinale* | + | + | + | + | + | + | + | + | + | + | + | + | + | + | + | + | + | + | + | + |
| *Nelumbo nucifera* Gaertn. |  | + | + |  | + |  |  | + | + |  |  | + |  |  |  |  |  |  |  | + |
| *Nuphar lutea* (L.) Sm. |  | + | + | + | + | + | + | + | + | + | + | + |  |  | + |  |  |  |  | + |
| *Nymphaea alba* L. subsp. *alba* | + | + | + | + | + | + | + | + | + | + | + | + |  |  | + | + | + | + |  | + |
| *Nymphaea alba* L. subsp. *minoriflora* (Simonk.) Asch. et Gr. |  |  | + |  |  |  |  |  |  |  |  |  |  |  |  |  |  |  |  |  |
| *Nymphaea mexicana* Zuccarini |  | + |  |  | + |  |  |  |  |  |  |  |  |  |  |  |  |  |  |  |
| *Nymphaea x marliacea* Wildsmith, pro sp. |  |  | + |  |  |  |  |  |  |  |  |  |  |  |  |  |  |  |  |  |
| *Nymphoides peltata* (S.G. Gmel.) Kuntze |  | + | + | + | + | + |  | + | + |  |  | + |  |  |  |  |  |  |  | + |
| *Oenanthe aquatica* (L.) Poir. |  | + | + |  | + | + | + | + | + | + |  | + | + | + | + | + | + |  | + |  |
| *Oenanthe javanica* DC. |  |  | + |  |  |  |  |  |  |  |  |  |  |  |  |  |  |  |  |  |
| *Ottelia alismoides* (L.) Pers. |  | + | + |  |  |  |  |  |  |  |  |  |  |  |  |  |  |  |  |  |
| *Paspalum dilatatum* Poir. |  | + | + | + | + | + | + | + | + | + | + | + | + | + | + | + | + | + | + | + |
| *Paspalum distichum* L. |  | + | + |  | + | + | + | + | + | + | + | + | + | + | + | + | + | + | + | + |
| *Paspalum quadrifarium* Lam. |  |  |  |  |  |  | + |  | + |  |  |  |  |  |  |  |  |  |  |  |
| *Persicaria amphibia* (L.) Delarbre | + | + | + | + | + | + | + | + | + | + | + | + | + | + | + | + | + |  | + | + |
| *Phragmites australis* (Cav.) Trin. ex Steud. subsp. *australis* | + | + | + |  |  | + | + | + |  | + | + | + | + |  | + |  | + |  | + |  |
| *Phragmites australis* (Cav.) Trin. ex Steud. subsp. *chrysanthus* (Mabille) Soják |  |  | + |  |  | + |  |  |  |  |  |  |  |  |  |  |  |  |  |  |
| *Pilularia minuta* Durieu ex A. Braun |  |  |  |  |  |  |  |  |  |  |  |  |  |  |  | + |  |  |  | + |
| *Pistia stratiotes* L. |  |  | + |  | + | + |  |  |  |  |  |  |  |  | + |  |  |  |  |  |
| *Plantago uniflora* L. |  |  | + |  |  |  |  |  |  |  |  |  |  |  |  |  |  |  |  | + |
| *Pontederia cordata* L. |  |  | + |  | + |  |  | + |  |  |  |  |  |  |  |  |  |  |  |  |
| *Posidonia oceanica* (L.) Delile |  |  |  |  | + | + | + |  | + |  | + | + | + |  | + | + | + | + | + | + |
| *Potamogeton acutifolius* Link |  | + | + |  | + | + |  | + |  |  |  |  |  |  |  |  |  |  |  |  |
| *Potamogeton alpinus* Balb. | + |  | + | + | + | + |  |  |  |  |  |  |  |  |  |  |  |  |  |  |
| *Potamogeton berchtoldii* Fieber | + | + | + | + | + | + |  | + |  |  |  | + | + | + | + |  |  |  |  |  |
| *Potamogeton coloratus* Hornem. | + |  |  |  | + | + |  |  | + |  |  |  |  |  |  | + | + |  |  | + |
| *Potamogeton compressus* L. |  |  |  |  | + |  |  |  |  |  |  |  |  |  |  |  |  |  |  |  |
| *Potamogeton crispus* L. | + | + | + | + | + | + | + | + | + | + | + | + | + | + | + | + | + | + | + | + |
| *Potamogeton filiformis* Pers. | + |  |  | + | + | + |  |  |  |  |  |  |  |  |  |  |  |  | + |  |
| *Potamogeton friesii* Rupr. |  |  | + |  | + | + |  |  | + |  |  |  |  |  |  |  |  |  |  |  |
| *Potamogeton gramineus* L. | + | + | + | + |  | + |  |  |  |  |  | + | + |  |  |  |  | + | + |  |
| *Potamogeton lucens* L. | + | + | + | + | + | + | + | + | + | + | + | + | + |  | + | + | + |  | + |  |
| *Potamogeton natans* L. | + | + | + | + | + | + | + | + | + | + | + | + | + | + | + | + | + | + | + | + |
| *Potamogeton nodosus* Poir. |  | + | + | + | + | + |  | + | + | + | + | + | + | + | + |  |  | + | + | + |
| *Potamogeton obtusifolius* Mert. & W.D.J. Koch |  |  | + |  |  |  |  |  |  |  |  |  |  |  |  |  |  |  |  |  |
| *Potamogeton pectinatus* L. | + | + | + | + | + | + |  | + | + | + | + | + | + |  | + | + | + | + | + | + |
| *Potamogeton perfoliatus* L. |  | + | + | + | + | + |  | + | + | + |  | + | + |  |  |  |  |  | + |  |
| *Potamogeton polygonifolius* Pourr. |  | + | + |  |  | + | + | + | + |  |  | + | + | + |  |  |  | + | + | + |
| *Potamogeton praelongus* Wulfen |  |  |  | + |  |  |  |  |  |  |  |  |  |  |  |  |  |  |  |  |
| *Potamogeton pusillus* L. |  | + | + | + | + | + | + | + | + | + |  | + | + | + |  |  |  | + | + | + |
| *Potamogeton schweinfurthii* A. Benn. |  |  |  |  |  |  |  | + | + |  | + |  |  |  |  | + |  |  |  | + |
| *Potamogeton trichoides* Cham. & Schltdl. |  | + | + |  | + | + |  | + | + |  | + | + | + |  |  |  |  | + | + | + |
| *Potentilla palustris* (L.) Scop. | + | + | + | + | + | + |  |  |  |  |  |  |  |  |  |  |  |  |  |  |
| *Ranunculus circinatus* Sibth. |  | + | + | + | + | + |  | + |  |  |  |  | + |  |  | + |  |  |  |  |
| *Ranunculus fluitans* Lam. |  | + | + |  | + |  |  |  |  |  |  |  |  |  |  |  | + |  |  | + |
| *Ranunculus lingua* L. |  | + | + | + | + | + |  | + | + | + |  | + | + | + |  |  | + |  |  |  |
| *Ranunculus omiophyllus* Ten. |  |  |  |  |  |  |  |  |  |  |  |  |  |  | + |  | + | + | + |  |
| *Ranunculus peltatus* Schrank subsp. *baudotii* (Godr.) C.D. Cook |  |  |  |  | + |  |  | + | + | + | + | + |  |  |  | + |  | + | + | + |
| *Ranunculus peltatus* Schrank subsp. *fucoides* (Freyn) Muńoz Garm. |  |  |  |  |  | + |  |  |  |  |  |  |  |  |  | + |  |  | + | + |
| *Ranunculus peltatus* Schrank subsp. *peltatus* | + | + |  |  |  | + |  |  |  |  |  | + |  |  |  |  | + | + | + | + |
| *Ranunculus penicillatus* (Dumort.) Bab. *subsp. penicillatus* |  |  | + |  |  | + |  |  |  |  |  |  |  |  |  |  |  |  | + | + |
| *Ranunculus penicillatus* (Dumort.) Bab. subsp. *pseudofluitans* (Syme) S.D. Webster |  | + | + | + | + |  |  |  |  |  |  |  |  |  |  |  |  |  |  | + |
| *Ranunculus reptans* L. | + | + | + | + | + |  | + |  |  |  |  |  |  |  |  |  |  |  |  |  |
| *Ranunculus rionii* Lagger |  |  |  | + | + |  |  |  |  |  |  |  |  |  |  |  |  |  |  |  |
| *Ranunculus trichophyllus* Chaix subsp. *eradicatus* (Laest.) C.D.K. Cook | + | + | + | + | + | + |  |  |  |  |  |  |  |  |  |  |  |  |  |  |
| *Ranunculus trichophyllus* Chaix subsp. *trichophyllus* | + | + | + | + | + | + | + | + | + | + | + | + | + | + | + | + | + |  | + | + |
| *Rorippa amphibia* (L.) Besser |  | + | + | + | + | + | + | + | + | + | + | + | + | + | + | + | + |  |  | + |
| *Rorippa anceps* (Wahlenb.) Rchb. |  | + | + |  | + |  |  | + | + |  |  | + |  | + |  |  |  |  |  |  |
| *Rorippa armoracioides* (Tausch) Fuss |  | + | + | + |  |  |  |  |  |  |  |  |  |  |  |  |  |  |  |  |
| *Rumex hydrolapathum* Huds. |  | + | + |  | + | + | + | + | + | + |  | + | + | + | + | + | + |  |  | + |
| *Ruppia cirrhosa* (Petagna) Grande |  |  |  |  | + | + |  | + | + |  | + | + | + | + | + | + | + |  | + | + |
| *Ruppia maritima* L. |  |  |  |  | + | + |  | + | + |  | + | + | + |  | + | + | + | + | + | + |
| *Sagittaria latifolia* Willd. |  | + | + |  | + | + |  | + |  |  |  |  |  |  |  |  |  |  |  |  |
| *Sagittaria sagittifolia* L. |  | + | + |  | + | + | + | + | + | + |  | + |  |  |  |  |  |  |  |  |
| *Salvinia molesta* D.S. Mitch. |  |  |  |  |  |  |  |  | + |  |  | + |  |  |  |  |  |  |  | + |
| *Salvinia natans* (L.) All. |  | + | + |  | + |  |  | + | + | + |  | + |  |  |  |  |  |  |  |  |
| *Saururus cernuus* L. |  |  | + |  |  |  |  |  |  |  |  |  |  |  |  |  |  |  |  |  |
| *Schoenoplectiella juncoides* (Roxb.) Lye |  | + |  |  |  |  |  |  |  |  |  |  |  |  |  |  |  |  |  |  |
| *Schoenoplectus carinatus* (Sm.) Palla |  |  |  |  | + |  |  |  |  |  |  |  |  |  |  |  |  |  |  |  |
| *Schoenoplectus lacustris* (L.) Palla | + | + | + | + | + | + | + | + | + | + | + | + | + | + | + | + | + | + | + | + |
| *Schoenoplectus litoralis* (Schrad.) Palla |  |  |  |  | + | + | + | + | + |  | + | + |  |  | + | + | + | + | + | + |
| *Schoenoplectus mucronatus* (L.) Palla | + | + | + | + | + | + |  | + | + | + | + |  |  |  |  |  |  |  | + | + |
| *Schoenoplectus pungens* (Vahl) Palla |  |  |  |  | + | + |  | + | + | + |  |  |  |  |  |  |  |  | + |  |
| *Schoenoplectus tabernaemontani* (C.C. Gmel.) Palla | + | + | + | + | + | + |  | + | + | + | + | + | + | + | + | + | + | + | + | + |
| *Schoenoplectus triqueter* (L.) Palla |  | + | + | + | + | + |  | + | + | + |  |  |  |  |  | + |  |  | + |  |
| *Scrophularia umbrosa* Dumort. subsp. *Umbrosa* |  | + | + | + | + | + | + | + | + | + | + | + | + | + | + | + | + | + | + | + |
| *Sium latifolium* L. |  |  | + |  | + | + |  | + | + |  |  |  | + |  | + |  |  |  |  |  |
| *Sparganium angustifolium* Michx. | + | + | + | + | + | + |  |  |  |  |  |  |  |  |  |  |  |  |  |  |
| *Sparganium emersum* Rehmann |  | + | + | + | + | + |  | + | + |  |  | + | + | + |  |  | + | + | + | + |
| *Sparganium erectum* L. subsp. *erectum* | + | + | + | + | + | + | + | + | + | + | + | + | + | + | + | + | + | + | + | + |
| *Sparganium erectum* L. subsp. *microcarpum* (Neuman) Domin |  |  |  |  |  |  |  |  | + |  |  |  |  |  |  |  |  |  |  |  |
| *Sparganium hyperboreum* Beurl. ex Laest. |  |  |  | + |  |  |  |  |  |  |  |  |  |  |  |  |  |  |  |  |
| *Sparganium natans* L. |  |  | + | + |  | + |  | + | + |  |  |  |  |  |  |  |  |  |  |  |
| *Sparganium neglectum* Beeby |  | + | + | + | + | + |  | + | + |  | + | + | + | + |  | + | + | + | + | + |
| *Spirodela polyrhiza* (L.) Schleid. |  | + | + | + | + | + | + | + | + | + |  | + | + |  |  | + |  | + | + | + |
| *Trapa natans* L. |  | + | + |  | + | + |  | + | + | + |  |  |  |  |  |  |  |  |  |  |
| *Trichophorum alpinum* (L.) Pers. | + | + | + | + | + | + | + | + | + |  |  |  |  |  |  |  |  |  |  |  |
| *Trichophorum pumilum* (Vahl) Schinz & Thell. | + | + | + | + | + |  |  |  |  |  |  |  |  |  |  |  |  |  |  |  |
| *Typha angustifolia* L. | + | + | + | + | + | + | + | + | + | + |  | + | + | + | + | + | + | + | + | + |
| *Typha domingensis* (Pers.) Steud. |  |  |  |  |  |  |  | + | + |  | + |  |  |  |  |  |  | + | + |  |
| *Typha latifolia* L. | + | + | + | + | + | + | + | + | + | + | + | + | + | + | + | + | + | + | + | + |
| *Typha laxmannii* Lepech. |  |  | + | + | + | + |  | + |  |  | + |  | + | + |  |  |  |  | + |  |
| *Typha shuttleworthii* W.D.J. Koch & Sond. |  |  | + | + | + | + |  | + | + |  |  |  |  |  |  |  |  |  |  |  |
| *Utricularia australis* R. Br. | + | + | + | + | + | + | + | + | + | + |  | + | + |  | + | + |  |  | + |  |
| *Utricularia bremii* Heer ex Koell. |  | + | + | + | + |  |  |  | + |  |  |  |  |  |  |  |  |  |  |  |
| *Utricularia minor* L. | + | + | + | + | + | + | + |  | + | + |  |  |  |  |  |  |  |  |  |  |
| *Utricularia stygia* G. Thor |  |  |  | + | + |  |  |  |  |  |  |  |  |  |  |  |  |  |  |  |
| *Utricularia vulgaris* L. |  | + | + |  | + | + |  | + | + |  |  |  |  | + |  |  |  |  |  |  |
| *Vallisneria americana* Michx. |  |  | + |  |  |  |  |  |  |  |  |  |  |  |  |  |  |  |  |  |
| *Vallisneria spiralis* L. |  | + | + | + | + | + |  | + | + | + |  | + |  |  |  | + |  |  |  |  |
| *Veronica anagallis-aquatica* L. | + | + | + | + | + | + | + | + | + | + | + | + | + | + | + | + | + | + | + | + |
| *Veronica anagalloides* Guss. |  |  | + | + | + | + |  | + | + |  |  | + | + | + | + | + | + | + | + | + |
| *Veronica beccabunga* L. | + | + | + | + | + | + | + | + | + | + | + | + | + | + | + | + | + | + | + | + |
| *Veronica catenata* Pennell |  |  | + |  | + | + |  | + |  | + | + |  | + |  |  | + | + |  |  |  |
| *Wolffia arrhiza* (L.) Horkel ex Wimm. |  | + | + |  | + | + |  | + | + |  |  |  |  |  |  |  |  |  | + |  |
| *Zannichellia obtusifolia* Talavera et al. |  |  |  |  |  |  |  |  |  |  |  |  |  |  |  |  |  |  | + | + |
| *Zannichellia palustris* L. subsp. *palustris* |  | + |  | + |  | + |  | + |  |  |  |  | + |  | + |  |  | + | + |  |
| *Zannichellia palustris* L. subsp. *pedicellata* (Wahlenb. & Rosén) Arcang. |  |  | + |  |  | + |  | + |  |  | + | + | + |  |  |  |  | + | + |  |
| *Zannichellia palustris* L. subsp. *polycarpa* (Nolte) K. Richt. |  | + | + | + | + | + |  | + | + | + |  |  | + |  |  |  |  |  |  |  |
| *Zannichellia peltata* Bertol. |  |  |  |  |  |  |  |  | + |  |  |  |  |  |  |  | + |  | + |  |
| *Zostera marina* L. |  |  |  |  | + | + |  | + | + |  | + | + | + | + |  | + | + | + | + | + |
