# Supplementary material for: Aquatic Plant Diversity in Italy: Distribution, Drivers and Strategic Conservation Actions
Source: Front Plant Sci. 2018 Feb 13;9:116. doi: 10.3389/fpls.2018.00116 (PMC5816802; doi:10.3389/fpls.2018.00116)
Supplement: Supplementary file 2 [file Table_2.docx]

Supplementary Material

Aquatic plant diversity in Italy: regional distribution, trends and drivers

**Rossano Bolpagni*, Alex Laini, Chiara Stanzani, Alessandro Chiarucci**

*** Correspondence:** Corresponding Author: rossano.bolpagni@unipr.it

# Supplementary Figures and Tables

For more information on Supplementary Material and for details on the different file types accepted, please see [here](http://home.frontiersin.org/about/author-guidelines#SupplementaryMaterial).

## Supplementary Tables

**Supplementary Table 2.** Pearson correlations between the climatic and environmental drivers quantified in the 20 Italian regions; and the correlations with the aquatic plant (AP), hydrophyte (HY), and non-hydrophyte (NH) regional composition (three lines at the bottom). Only significant (P<0.05, in bold) and marginally significant (0.5≤P<0.1) correlations were shown. Square brackets identified the variables excluded from analyses due to large collinearity. Abbreviations are as follows: x = longitude; y = latitude; Area = area of a given region; Lake = regional total surface occupied by lakes; Rive = the total regional linear development of natural hydrosystems; Rain = regional mean annual precipitation; Aqre = regional aquifer recharge rate; Revt = regional mean annual real evapotranspiration; Temp = regional mean annual temperature; Altr = regional altitude range; H = regional heterogeneity index by Chappuis et al. (2012); Hidr = hydro-ecoregions heterogeneity; Inkm = regional inhabitants per km^2^.

|  | **[x]** | **[y]** | **Area** | **Lake** | **Rive** | **[Rain]** | **[Aqre]** | **[Revt]** | **Temp** | **[Altr]** | **H** | **[Hidr]** | **Inkm** |
| --- | --- | --- | --- | --- | --- | --- | --- | --- | --- | --- | --- | --- | --- |
| **y** | **-0.63** |  |  |  |  |  |  |  |  |  |  |  |  |
| **Area** |  |  |  |  |  |  |  |  |  |  |  |  |  |
| **Lake** |  |  | **0.66** |  |  |  |  |  |  |  |  |  |  |
| **Rive** |  |  | **0.89** | **0.60** |  |  |  |  |  |  |  |  |  |
| **[Rain]** | -0.42 | **0.75** |  |  |  |  |  |  |  |  |  |  |  |
| **[Aqre]** |  |  | **0.72** | **0.79** | **0.74** |  |  |  |  |  |  |  |  |
| **[Revt]** | **0.63** | **-0.95** |  |  |  | **-0.76** |  |  |  |  |  |  |  |
| **Temp** | **0.61** | **-0.93** |  |  |  | **-0.82** |  | **0.97** |  |  |  |  |  |
| **[Altr]** |  | **0.55** |  |  |  | **0.67** |  | **-0.66** | **-0.69** |  |  |  |  |
| **H** |  |  |  |  |  | **0.52** |  |  |  |  |  |  |  |
| **[Hidr]** |  |  |  | 0.38 |  | 0.38 |  |  |  |  | 0.40 |  |  |
| **Inkm** |  |  |  |  |  |  | 0.43 |  |  |  |  | **0.62** |  |
| **AP** |  |  | 0.70 | **0.77** | 0.71 |  | **0.87** |  |  |  |  |  |  |
| **HY** |  |  | **0.74** | **0.91** | 0.70 |  | **0.88** |  |  |  |  |  |  |
| **NH** |  |  |  | 0.70 |  |  | **0.75** |  |  |  |  |  |  |
